# What social and environmental considerations are important for socially assistive robotic adoption for pre-frail older adults at home: a scoping review, life cycle assessment and survey

**DOI:** 10.1186/s12877-025-06892-8

**Published:** 2026-02-27

**Authors:** Lucie Nield, Charlotte Jackson, Raphael Ricardo Zepon Tarpani, Matthew Story, Alessandro Di Nuovo, Sally Fowler-Davis

**Affiliations:** 1 University of Sheffield, Sheffield Centre for Health and Related Research (SCHARR), Sheffield, S1 4DA UK; 2 Sheffield Hallam University, City Campus, Sheffield, S1 1WB UK; 3 Department of Mechanical, Aerospace and Civil Engineering, Tyndall Centre for Climate Change Research, University of Manchester, Floor 5, Core 1 W, Engineering Building A, Booth Street E, Manchester, M13 9PL UK; 4 Anglia Ruskin University, Cambridge, UK

**Keywords:** Technology adoption, Socially assistive robotics, Older adults, Life cycle assessment

## Abstract

**Background:**

A complex evaluation of robotic adoption for older adults is necessary, given the pressing need to prevent ill health and disability. Critical thinking that incorporates an understanding of sustainability is needed for the future, if assistive robots are to be widely used. This research was undertaken as part of I’MACTIVE, a project that combined emergent technologies for older adults at risk of becoming frail. The aim was to analyse the sustainability of emerging socially assistive robotic technology for use with community dwelling older people and to assess the likelihood of adoption and acceptability.

**Methods:**

The study includes a scoping review of the social and environmental impacts of socially assistive robotics, a life cycle impact evaluation of a robot and potential metrics for assessing its circularity, and a subsequent survey in two stages of the perspectives of older adults on assistive robotics. The evaluation sought to assess the likelihood of adoption, given priorities and perspectives of older people. Framework analysis was used to combine findings.

**Results:**

The review included eight studies, but none addressed the social and environmental features of the robotic implementation in terms of needs of older people. Theories of adoption and acceptance do not yet include life cycle assessments of technologies. The environmental assessment of the TurtleBot4 robot indicated that its global warming potential is estimated at 68–118 kg CO_2_-eq. The most important components were the printed circuit boards and batteries, together responsible for over 60% of the impacts. The survey demonstrates that older adults are willing to use socially assistive robotics if robots have an appropriate level of function, cost, environmental impact and legal risk.

**Conclusions:**

Socially assistive robotics may benefit some older people but currently the design process inconsistently factors in the values and choices of older people. Nor does it consider the environmental implications of scaling the deployment of robotics in personal residences. Robots have the potential to help with the caregiving and domestic needs of the growing ageing population but need to be sustainably produced and have tangible functional benefit, identified by older people, to be acceptable.

**Supplementary Information:**

The online version contains supplementary material available at 10.1186/s12877-025-06892-8.

## Background

Health and health promoting activities in older adults are focussed on the prevention of ill health and disability in later life, and the promotion of better quality of life outcomes. However, there is variable engagement with health promotion activities often depending on perceived risk of developing specific conditions or becoming frail. Frailty is common in older adults and is defined as *“a distinctive health state related to the ageing process in which multiple body systems gradually lose their in-built reserves.”* [1] Physical and cognitive frailty are both conditions associated with older adults and providing successful interventions before people become frail (i.e. pre-frail) is known to be effective [2–4].

Assistive robots (ARs) have been defined as *“devices designed to help individuals with physical or cognitive limitations perform tasks, supporting daily activities, improving mobility, providing rehabilitation, and enhancing independence and quality of life*” [5]. Socially-assistive robotics (SARs) are designed to support users with social interactions rather than physical tasks [6]. In recent years, robotic and autonomous devices such as the Roomba vacuum cleaner, or Ring doorbell have provided innovation for domestic use from cleaning to communication, safety and companionship. Whilst these devices are largely perceived as luxury or novelty items, there is promise that they could have wider transformative benefits in domestic life [5]. SARs demonstrate many opportunities to assist older adults to live longer and more independently at home [6, 7]. Robots equipped with advanced artificial intelligence (AI), sensors and social interaction capabilities offer promising tools to address the challenges of cognitive decline in the elderly [8]. Digital innovation and robotic design in health and care has so far been driven by the commercial sector, in part to sustain health and in part to mitigate healthcare risks and needs but with industry motivations to profit and to sustain business. Whilst these are legitimate goals, they may be at odds with the additional requirements to reduce costs and to disinvest in high carbon supply chains. Widespread adoption of SARs is still lacking, indicating a lack of understanding of the factors and mechanisms driving consumer acceptance [9].

For several decades technology adoption models have been used to signal the importance of understanding the human and behavioural factors in relation to using new technology [10]. The innovation process recognises that the needs and acceptance of individuals is a critical dimension of design and development [11]. Adoption of technologies therefore also requires an investigation of acceptance as a means of understanding macro-resistance [12]. Success of robotic adoption in the domestic setting has been less than anticipated [13] due a perception of robots as enablers to menial tasks, which effectively limits the opportunity for the ‘social’ in socially assistive robotics.

The design and deployment of assistive robotic devices is predicated on the collaboration with older people in terms of user-centred design [14] specifically how human operators manage and interact with machines and how they are supported by adaptable interfaces, simulations and real-time data collection and analysis. However robotic designs are often limited by specific technical features or resource limitations that reduce the degree of flexibility [15] and this necessitates a comprehensive examination for the purpose of improving the device for the benefit of older users.

There is a need to develop a more critical approach and raise awareness of the implementation and sustainability of enabling technologies and to build this understanding into the design process. Digital technologies and AI are widely regarded as essential to transformational activities across sectors [16]. Adoption processes have been oversimplified particularly in a context in which accumulated digital data have increasing cost to the environment and commercial value [17]. There are therefore significant topics to explore in relation to how digital data and the algorithms that collect and manage these data contribute to both health benefit and risk.

One way in which issues are commonly explored, is by using researcher-developed robots such as Pepper and NAO which aim to assist with basic communication, companionship and social interaction needs. In this study, we used a TurtleBot4 robotic platform as an example of a low cost home robotic device which has the potential to be adapted to meet older adult needs. The TurtleBot4 is a basic open-source development robot comprising of a robotic iRobot Create3 mobile vacuum cleaner base, Raspberry Pi 4 software and AI stereo camera [18] which can be easily adapted to host and integrate additional software or devices such as iPads, additional sensors and voice recognition software.

It is important to consider how the circular economy (CE) can promote and mitigate the environmental impact of digital devices [19]. For instance, CE often seeks to promote the remanufacturing of electric and electronic equipment (EEE) to increase the circularity of plastics and metals (and reduce environmental impacts originated from their production) or increasing resource efficiency by designing EEE with longer lifespans. Given the significant challenge of climate change and the dependence of robotic devices on electricity and battery power, there is now a need to test the functionality of the new devices within an environmental context. To isolate the cost/value of component parts and ultimately approximate the carbon footprint of devices is important, not least because of initiatives such as the Greener National Health Service programme [20] in England and the ambition to achieve a triple bottom line [21] in relation to cost, quality and environmental sustainability.

There is a gap in the understanding of what acceptability looks like for older adults, and how robotic implementation could be sustained. The aim was to analyse the sustainability of emerging socially assistive robotic technology for use with community dwelling older people and to assess the likelihood of adoption and acceptability. The research was undertaken as part of I’MACTIVE (https://www.shu.ac.uk/advanced-wellbeing-research-centre/projects/im-active), a project that combined emergent technologies for older adults at risk of becoming frail. By analysing the sustainability of emerging robotic technology through literature, life cycle assessment and survey. Recommendations for additional design criteria are included given the pressing need to prevent ill health and disability and to refine the specifications for long term adoption.

## Methods

A multi-methods approach was used to understand factors leading to sustainability or conversely presenting a barrier to sustained implementation, which enabled an assessment of robotic design against priorities of older adults alongside the life cycle and adoption criteria associated with socially assistive robotic development. The study consisted of three parts, starting with a scoping review of the literature that supports two further investigations. The literature focuses on the adoption of home socially assistive robotics for older adults and seeks to identify the main drivers to motivate and understand older adults’ adoption. Then dissection and life cycle analysis of the TurtleBot4 [18] for an experimental assessment of a platform, widely used in research and development of robotics and finally the survey with older adults who may be at risk of becoming frail and whose views, opinions and likelihood of robotic adoption at home is critical.

The research was carried out by health care professional and public health researchers (LN, SFD), computer scientists, robotic specialists and lifecycle analysis engineers (RT, MS, AD) and research assistants with a background in psychology.

### Scoping review

The scoping review was carried out using Arksey and O’Malley’s framework [22] that is particularly suited to projects where literature is used as a benchmark for new topics and ones where policy, practitioners and service users are stakeholders. We followed the 5-stage process of Stage 1: identifying the research question; Stage 2: identifying relevant studies; Stage 3: study selection; Stage 4: charting the data and Stage 5: collating, summarizing and reporting the results. The larger project (I’MACTIVE) considered the factors which should be considered when designing technology and robotics for pre-frail older adults living in the home. This scoping review involved a systematic search with a framework analysis that allowed researchers to produce a pragmatic assessment of the existing data [23] related to the aim of analysing the sustainability of emerging robotics with a view to enabling greater adoption. The scope of this literature review was to understand the social and environmental/climate-related considerations which are important for robotic adoption for pre-frail older adults at home. The Arksey and O’Malley framework guidelines suggest the use of PRISMA in reporting findings and permit limits to searches as justified by policy and practice rationale [22].

#### Search strategy

The search strategy for this scoping review was developed within the research team with the aim of identifying all studies that measured social and environmental consideration for adoption of domestic assistive health and care robotics in older adults (See Search Strategy, Appendix 1). By environmental considerations, we include recyclability, carbon footprint, water usage, energy usage, energy efficiency, energy generation, clutter/waste management, use of renewable materials, reuse, replacement of parts and cost-value. Databases were searched from 2008 when smartphone technology became commonplace to February 2024. Searches including Medline, Google Scholar, Web of Science, CINAHL and Scopus. Identified papers were imported into Covidence software for screening, with any additional manually searched papers added if deemed potentially relevant.

#### Inclusion/exclusion criteria

Studies were included if they evaluated robotics in the context of older adults who were home-based, community dwelling participants including those living in sheltered accommodation, and where results included a measure of adoption of robotics technology. All studies were in the English language.

Studies were excluded if they reported non-human or child participants, in surgical, secondary care, palliative care, or care homes/care environments. Studies in a language other than English were excluded.

However, due to the broad nature of the review, we did not limit by study design or specific outcome measures. Additionally, studies were not excluded based upon their quality as quality assessment is not a priority in scoping reviews.

#### Title and abstract screening

Covidence software was used to complete title and abstract screening of original studies. Duplicates were removed followed by any conference abstracts, editorials, book and commentaries which were excluded at this screening stage (see PRISMA; Fig. 01). Screening of the literature was completed by one reviewer (CJ), with 20% of title and abstracts checked by another reviewer (LN) for agreement. Conflicts were managed and agreed by discussion between the reviewers.

#### Full text screening

Covidence software was also used to complete the full text screening. Studies which reported on older adults, living in their home and describing robotics technology adoption were included for data extraction. Full text screening was completed by two independent reviewers (CJ and LN), with conflicts managed by discussion to achieve a consensus, or by a third reviewer if necessary (SFD).

#### Data extraction and analysis

A Microsoft Excel form was used to collate the data extracted from each article (see Appendix 2), in line with the inclusion criteria: Authors, Publication Date, Country, Aim of study, Study design, Research funder, Method of recruitment, Population description, number of participants. Information on how social and environmental factors were measured in the following categories were included: Acceptability/Adoption measures, Usage/Useability measures, Sustainability measures, and Opinions and attitudes. This provided a baseline for the assessment of the overall findings, prior to data synthesis. Data was then synthesised using Framework Analysis and grouped within themes by the authors (CJ, LN) & SFD to identify the type and availability of research data relevant to the scope of the research.

### Life cycle assessment and circular economy

Additionally, and aligned to the aim to analyse the sustainability of robotics for use with community dwelling older people an investigation of the life cycle impacts of a TurtleBot4 device was undertaken. The sustainability of parts, components and energy consumption was highly relevant to the assessment of adoption for home-dwelling deployment of robotics. Life cycle assessment evaluates the operation, maintenance, and disposal of this type of assistive device. The TurtleBot is a robotic learning platform [18] not intended to be a socially assistive or healthcare robotics device but widely used in research and for application development. The purpose was therefore to assess the sustainable features of the device and analyse the features that can be made more robust in relation to manufacture. The complete disassembly of the device was followed by meticulously evaluating the dismantling difficulties and weighing its components. By systematically deconstructing the device, we aimed to gain insights into the intricacies of its design and the materials/components embedded within it [24]. Following the disassembly, the focus shifted to assessing the device's energy consumption. This involved measuring electricity consumption for battery charge through a monitoring device and estimating it based on the operating time and technical specifications from the manufacturer's manual. By quantifying the materials and energy consumption associated with the TurtleBot4 device, we were able to have an overview of the environmental impacts during its life cycle. This estimation was conducted using the latest ReCiPe v1.03 midpoint (H) impact methodology [25] and ecoinvent v3.1 [26] to identify environmental hotspots and potential areas for improvement. A total of five impact categories were chosen due to them being the most common in life = cycle environmental studies: global warming potential (in kg CO2-eq.), fossil fuel resource potential (in kg oil-eq.), surplus ore potential (in kg Cu-eq.), freshwater ecotoxicity potential and human toxicity-non cancer potentials (both in kg 1,4-dichlorobenzene (DB)-eq.).

#### Surveys of older adults

The second investigation, linked to the main aim, focussed on the potential sustainability factors for community dwelling older people and to assess the likelihood of adoption and acceptability. The surveys were designed to understand the acceptance of socially assistive and assistive robotics in older adults and was developed by researchers and piloted with a group of five older adults. The surveys were adapted in response to their feedback before being circulated to older adults via an online Qualtrics survey through the Collaborative Older Adults Research Network (COARN) in South Yorkshire. COARN was established by a group of multidisciplinary researchers at Sheffield Hallam University and consists of older adults and key stakeholders who advocated for, or worked closely with older adults throughout the South Yorkshire Mayoral Combined Authority. The survey aimed to recruit adults who may have lived experience of, or advocate for older adults in the network and beyond. Typically, COARN members are community-dwelling older adults who live in the region, voluntary sector or NHS organisations and local authority partners, The survey questions addressed preferences for, and interest in, current and future use of electronic and robotic devices, and any environmental considerations, with a particular focus on ‘robots that can assist with physical mobility and occupational therapy type tasks’. The first survey was designed to capture current behaviours and engagement of older adults with familiar domestic electrical, technological and robotic equipment in daily use in most homes. This was to create a baseline of environmental or energy usage concerns within the older community dwelling population. The second survey was sent out 6 weeks later to all those who provided their contact details and gave consent to receive it after completion of the first survey. The second survey was based on the Unified Theory of Acceptance and Use of Technology (UTAUT) framework which examines whether technology is accepted based on the effects of four factors: performance expectancy, effort expectancy, social influence and facilitating conditions [27] where participants were asked to rate their agreement to statements using a Likert scale 1–7 (with 4 being in the middle).

The study was approved by the university research ethics committee (ER62022396) and all participants provided informed consent prior to completing the questionnaires. Data from the life cycle analysis of the TurtleBot4 was included in the second survey and compared to familiar devices for energy usage comparisons. Overall, the surveys evaluated their perceptions of health, care and socially assistive robots and factors relating to acceptance and usage of such technologies.

## Results

### Summary of findings: scoping review

#### Included papers

**Fig. 1:**
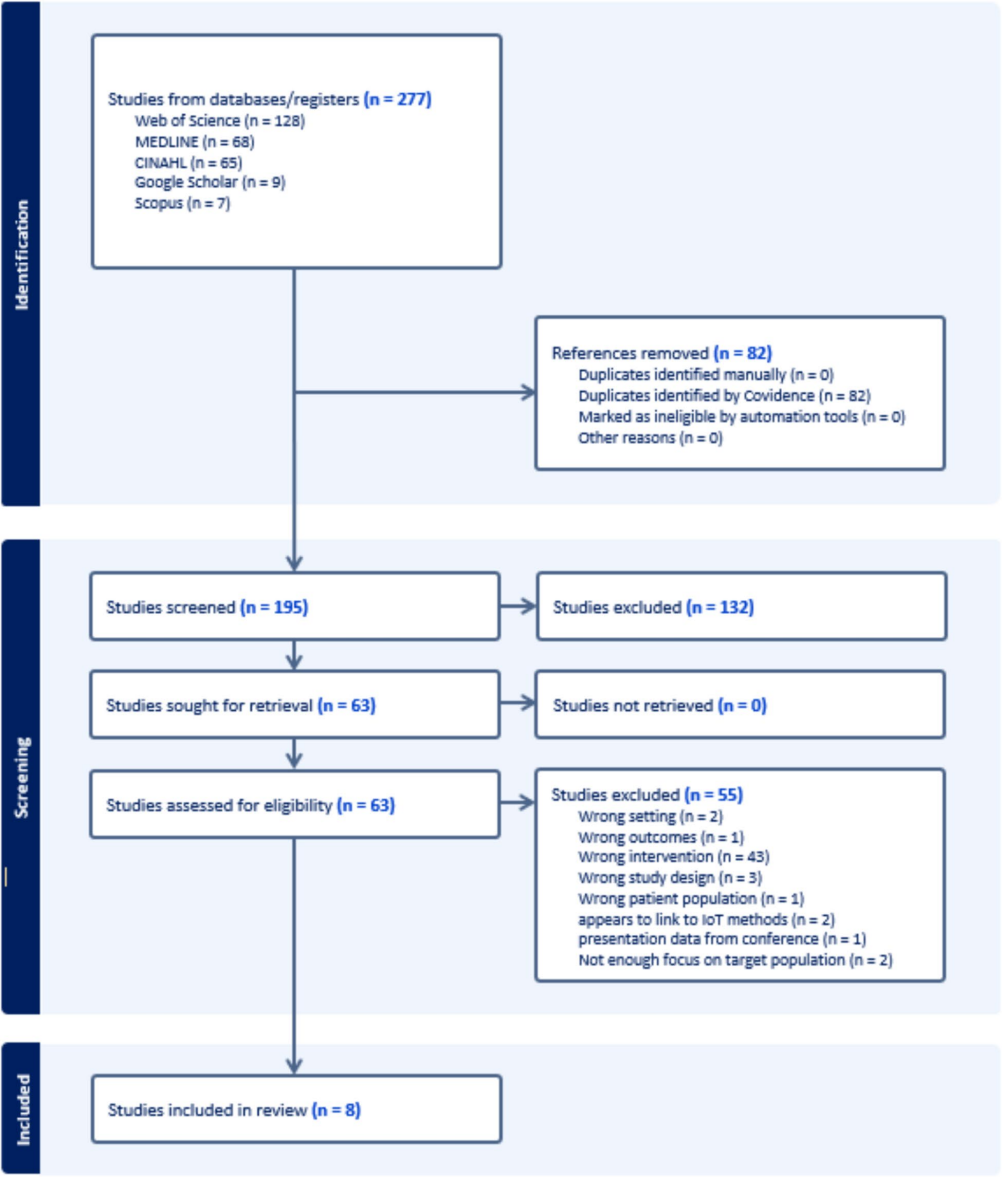
PRISMA diagram

In total, 277 papers were identified by the search. Following screening and data selection, 8 papers were identified for data extraction presented in the PRISMA diagram (Fig. 1) which related to the research aim.

Eight papers were selected which were published between 2011 to 2024, representing research from the United States of America (*n* = 4), China (*n* = 1), Poland (*n* = 1), UK (*n* = 1) and France (*n* = 1). The selected articles alongside their demographic characteristics and measures of social and environmental factors are presented below in Table 1. In total, these studies represented the views of 614 older adults.

**Table 1:** Summary of papers

Title	Author(s) and year	Country	Participants	Aim	Summary of Main Findings
Perspectives on a telepresence robot at an independent living facility: Lessons learned and implications	Arthanat, S., Rossignol, H., Preble, E., Grimm, K., Corvini, M., Wilcox, J., Aytur, S. and Doyle, M. (2024) [28]	USA	N = 10	To gather the perspectives of residents, trainers and staff at a retirement facility on their experience with a telepresence robot during and following a five-week wellness program	Incremental exposure to the telepresence robot helped to bridge any gaps in technology literacy, increasing confidence. User acceptance enabled the facilitation of social connections, aging in place and the delivery of health services. However, older adults still favoured the delivery of in person healthcare services and support
Mobile remote presence systems for older adults: Acceptance, benefits and concerns	Beer, J. M. and Takayama, L. (2011) [29]	USA	N = 12	To understand older adults' views on what a mobile remote presence (MRP) system may be used for and to understand older adults perceived benefits and concerns about the system	The older adults' opinions of the MRP system were overall positive in nature (66%). The identified benefits of using the system (n = 174) significantly outweighed any related concerns (n = 124), χ^2^ = 15.4, p <.0001. When the benefits of using technology are clear, older adults are willing to adopt its usage
Acceptance and perceived usefulness of robots to assist with activities of daily living and healthcare tasks	Hall, A. K., Backonja, U., Painter, I., Cakmak, M., Sung, M., Lau, T., Thompson, H. J. and Demiris, G. (2017) [30]	USA (Seattle, Washington Metropolitan Area)	N = 499	To understand acceptance and perceived usefulness of tasks performed by robots among young, middle-aged, and older adults	Robots have the potential to help with the caregiving and domestic needs of the growing ageing population and older adults with multiple chronic conditions. However, less than half (40.2%) of older adults disclosed that robot assistance in the hospital would be acceptable or wanted (p < 0.001)
Are we ready for robots that care for us? Attitudes and opinions of older adults towards socials assistive robots	Pino, M., Boulay, M., Jouen, F. and Rigaud, S. A. (2015) [31]	France (Paris)	N = 25	To clarify several aspects related to the acceptance of Socially Assistive Robots (SAR) by older adults. Also, examining if opinions and attitudes toward SAR differed among three groups of community living older adults	It is a complex task to ensure that the design of SARs are both acceptable and efficient, systems need to be flexible and customisable to adapt. Elderly people concerned by cognitive impairment recognise the potential of SARs for supporting their health and social care at home
Robots for elderly care: Review, multi-criteria optimization model and qualitative case study	Sawik, B., Tobis, S., Baum, E., Suwalska, A., Kropinska, S., Stachnik, K., Perez-Barnabe, E., Cildoz, M., Agustin, A. and Wieczorowska-Tobis, K. (2023) [32]	Poland (Krakow)	N = 12	To perform focus group discussions to collect opinions about robot-related requirements of the elderly	Before introducing the robot to the older person, pre-training should be given that considers a wide range of ethical and practical issues. It is essential to involve the future robot users in preparation and customisation, following the needs and preferences of the older people themselves
Robot companions and sensors for better living: Defining needs to empower low socio-economic older adults at home	Vagnetti, R., Camp, N., Story, M., Ait-Belaid, K., Bamforth, J., Zecca, M., Di Nuovo, A., Mitra, S. and Magistro, D. (2024) [33]	UK	N = 17	To analyse and understand the perception and needs of low-income older adults regarding SARs, monitoring technologies and there use in home	Five key areas were highlighted that should be considered when designing SARs: 1) Promote and monitor an active lifestyle 2) aid with daily errands and provide physical assistance 3) reduce loneliness and isolation 4) considerations relating to monitoring technologies 5) barriers impacting SAR acceptance and usage
Technology to support aging in place: Older adults' perspectives	Wang, S., Bolling, K., Mao, W., Reichstadt, J., Jeste, D., Kim, H. C. and Nebeker, C. (2019) [34]	USA (San Diego, California)	N = 31	To involve the residents of a local senior housing community in conversations about technologies that might facilitate their continued independent living status	The potential benefits offered by technologies were often prevented due to older adults having a lack of confidence in their ability to understand how to use or access them. Also, inadequate software and hardware interfaces cause frustration in older adults' interactions with digital products
Older adults actual use and adoption intention of smart health technologies in Hong Kong	Zhang, J., Wang, H., Lee, B. Y. H., Pang, M. Y. C. and Luximon, Y. (2022) [35]	China (Hong Kong)	N = 8	To investigate if the use of smart health care technologies, participants health status, sensation, and capability could be the motivators of actual use and adoption intention	Nursing and Assistive Robots (NARs) had not been used by any of the participants. However, 62.5% of participants stated that they would be willing to use them (mean = 6.20). Results suggest that gender, age, living situation, cognitive capabilities and educational level can all influence adoption intention

#### Summary of scoping review

The selected studies highlight several key themes including robotic design; appearance and functionality; technology literacy and implementation; acceptance and perceived benefits; barriers to acceptance and concerns; and social connections. However, it is important to state that the themes that have been identified from the literature, fail to include any mention of sustainability, climate considerations, supply chain issues linked to environment and net zero purchasing in the health and social care sector, or the environmental costs of utilising, maintaining, upgrading and disposing of robotic and sensor devices.

##### Robotic design, appearance and functionality

Relating to acceptance, design preferences emerged as a critical factor. Pino et al. (2015) found that older adults tended to be more accepting of animal-like robots as opposed to those with a human-like appearance [31]. This was consistent with research by Sawik et al. (2013), who highlighted that participants’ preferred robots that had a ‘machine like appearance’ [32]. It was also considered essential for robots to be both customisable and flexible in design, with participants most favoured functionalities being (a) cognitive support applications to compensate cognitive impairment (e.g. task reminding, locating lost items); (b) communication services to keep an active social life (e.g. video calls, email); (c) risk prevention and healthcare applications (e.g. falls detection, management of critical situations); and (d) applications to support daily living tasks (e.g. online grocery shopping, journey planning, simplified internet access). Additionally, monitoring and reminders were considered useful tools. Furthermore, a critical safety aspect was highlighted, suggesting that socially assistive robots could potentially detect any risky situations and contact the emergency services following a fall. This was considered particularly useful for older people living alone.

##### Technology literacy and implementation

Research by Wang et al. (2019) identified that a lack of technology literacy and confidence in using modern devices prevented older adults from fully embracing the technologies that were available to them [33]. It was suggested that providing static instructional materials, such as printed manuals would aid older adults who typically favoured these formats over online resources. Improving interface designs to accommodate physical declines, such as visual impairment, and using familiar technological vocabulary were also suggested.

##### Acceptance and perceived benefits

Many studies report that in general, older adults are receptive to the adoption of robotic technologies. Research by Beer and Takayama (2011) identified that 66% of older adults had positive opinions about a mobile remote presence (MRP) system which was valued for its ability to reduce the need for travel and enhance socialisation opportunities [29]. The identified benefits of using the system (*n* = 174) significantly outweighed any related concerns (*n* = 124), χ^2^ = 15.4, *p* < 0.0001. Participants identified that being able to see the person on the other side of the MRP (visualisation) was the most favoured benefit. They also expressed a preference to have the ability to control the system independently rather than relying on others to control it. Similarly, Pino et al. (2015) noted that older adults appreciate functionalities such as cognitive support, risk prevention, communication services, and assistance with daily living tasks provided by SARs [31]. These technologies were understood to enhance older adults' ability to stay connected and manage their daily activities more effectively. However, it was clear that while older adults recognised the benefits these systems may provide, robotic devices should be used to supplement in-person health care and not replace it. Relating to adoption intention, Zhang et al., (2022) highlighted that gender, age, living situation, cognitive capabilities and educational level can all have an influence [35]. It was reported those who expressed the strongest adoption intention were aged below 75, male, higher educated, of better health and with good cognitive capabilities. Also, those living with family reported a higher adoption intention than those living alone. It was suspected that this may be due to potential support provided by families when using new technologies.

##### Barriers to acceptance and concerns

Despite the identified benefits mentioned above, many concerns and barriers to robotic adoption were also identified. Vagnetti et al. (2024) highlighted financial concerns and the complexity of using SARs as significant barriers [34]. Hall et al. (2017) found that older adults were less comfortable with robots performing tasks such as medical operations, with less than half (40.2%) of participants identifying this as something they would find acceptable (p < 0.001) [30]. Other identified concerns and barriers by Beer and Takayama (2011) were privacy issues, the etiquette of managing calls, and a lack of face-to-face interaction [29].

##### Social connections

The social implications of adopting these technologies are significant. It was highlighted that systems such as the MRP and SARs can enhance social connections by facilitating communication with family and friends, therefore reducing loneliness and social isolation. Beer and Takayama (2011) and Vagnetti et al. (2024) noted that these technologies could provide valuable social support especially for those living alone [29, 34]. However, there is a strong preference for these technologies to complement rather than replace human interactions. It was also suggested that when the benefits of using technology are clear; independently living, healthy older adults are more willing to adopt usage in both social and care contexts. Participants in the research by Pino et al., (2015) recognised the potential of using SARs within the home to enhance their health and social care provision [31].

#### Summary

In summary, while it is clear that older adults recognise the potential benefits of robotic and technological assistance, successful adoption is reliant on addressing privacy concerns, cost barriers, design preferences, and the need for training and support. Enhancing technological literacy and ensuring these technologies complement human interactions can significantly improve their acceptance and effectiveness in supporting ageing in place, and elements of health, assistance and care delivery. The studies explored within this scoping review highlight the importance of considering both the practical and social dimensions of technology use among community dwelling older adults to enhance their overall quality of life. However, more research needs to be done to address sustainability and environmental concerns as this is something that is not currently included in any of the literature explored.

##### Summary of findings: life cycle assessment

The TurtleBot4 device was assessed for key life cycle requirements in order to estimate the wider environmental impact for adoption. It requires approximately 240 min and 0.10 kWh to fully charge its battery, which lasts between 20 to 60 min per charge. Based on an operational frequency of either daily or weekly use, and considering the battery's lifespan of 300 cycles, the TurtleBot4 is expected to have a total lifespan of around 5.8 years – thus this period could necessitate the use of 1 to 5 batteries. The total weight of the device is estimated between 4,521 and 5,461 g, predominantly composed of plastics (54–65%), with significant contributions from batteries (5–22%), printed circuit boards (9–11%), stainless steel (8.5–10.5%), and aluminium (7.5–9%). The energy consumption over its lifespan is projected to be between 30 to 150 kWh. Combining materials and energy consumption, the life cycle of the device has a global warming potential of 68 to 118 kg CO2-eq., and a fossil fuel resource impact ranging from 19 to 35 kg oil-eq.

The findings from the environmental assessment highlighted that the most significant environmental impacts stem from the printed circuit boards and batteries, accounting for over 60% of the total impacts in the categories assessed. Regarding CE strategies, to mitigate these effects, it is advisable to take actions to reduce the frequency of battery replacements as much as possible and ensure the sourcing of batteries from environmentally responsible suppliers. Proper disposal and recycling methods, particularly within the UK, should be prioritized to recover valuable metals from the printed circuit boards and batteries [36–38]. Additionally, redesigning components for the use or materials with lower environmental impacts and easier disassembly for repairing and recycling could further enhance the device's sustainability [39]. For instance, substituting aluminium parts with plastic ones and improving the accessibility of internal components to simplify battery replacement. These considerations are integral to the circularity evaluation of electronic devices and warrant further exploration in subsequent studies.

##### Summary of findings: survey

Surveys were sent via the Collaborative Older Adult Research Network (COARN) in South Yorkshire. One hundred participants responded to the first survey, and forty-six people responded to the second questionnaire which evaluated their perceptions of health, care and socially assistive robotics and factors relating to acceptance and usage of such technologies. Most respondents were White British, female, and in generally good self-reported health (Table 2). Additionally, participant characteristics demonstrate that some respondents had physical and mental health needs indicating that overall, the group of respondents were an appropriate ‘pre-frail’ population. In addition, many were of higher educational status, and replied promptly to an online survey received via e-mail implying that they were already confident users of technology and had good accessibility to electronic devices. The diversity of the ethnicities of the sample group was not representative of a Yorkshire and Humber population which is typically 80.9% non-white British [40], however this was appropriate for the preliminary study and provided indicative data.

Participants reported that only 11% would perceive themselves or someone they care for as being frail according to the British Geriatric Society definition of, “Frailty is a distinctive health state related to the ageing process in which multiple body systems gradually lose their in-built reserves.” [1]. The rich descriptions generated via the survey data was intended to generate insights into the older adult, pre-frail perspective. The data was averaged and trends identified (Table 2).

**Table 2:** Participant characteristics of survey respondents from survey 1 (*n* = 100)

Gender (%)	Male	32
	Female	68
Age (%)	65–74	61
	75–84	33
	85 +	2
Ethnicity (%)	White	96
	White Irish	1
	Black/Black British Caribbean	2
	Prefer not to say	1
Learning disability (%)	Yes	1
	No	99
Mental Health issues (%)	Yes	2
	No	98
Physical Health issues (%)	Yes	7
	No	88
	Not sure/prefer not to say	5
Overall self-reported health rating (%)	Very good	24
	Good	46
	Average	24
	Poor	3
	Very poor	1
	Prefer not to say	2
Highest level of education (%)	Secondary school	9
	College	15
	University	41
	Postgraduate study	34
	Prefer not to say	1

#### Current technology usage

Study respondents owned a variety of electrical equipment, which was used at differing frequencies. The most commonly owned and frequently used electronic items were mobile phones (86% used daily), televisions (81% used daily) and kettles 87% used daily). Additional items that people expressed a desire for were robot hoovers (22%), electric blankets (18%), air fryers (15%) and dishwashers (15%). However, 56% of individuals did not want a robot hoover.

The majority (85%) of individuals reported that they use all electrical items and technology independently and without the need for support from friends and family. 7% already received support to use items, and 3% didn’t currently access support but would like further assistance to use their devices.

#### Financial and environmental barriers to usage

Of 100 respondents, 54% were experiencing high levels of concern regarding their energy bills, with 36% responding that they were sometimes concerned. 49% felt that they had constantly worked hard to reduce their energy consumption in the home, and 42% felt that they had sometimes worked hard to reduce their energy consumption. 51% reported that they were constantly concerned about their environmental impact, with 42% reporting that this was sometimes of a concern to them. Despite this, only 23% consistently turned off electrical equipment overnight with 49% reporting that they sometimes did this. However, 41% always looked for low energy usage models when purchasing new technology and electrical equipment, and 44% only sometimes considered this.

Generally, respondents reported that they only replace their electrical equipment when it finally dies (79%), but 13% upgraded to a smarter or more technical model. A small number of respondents reported that they only replaced equipment when they could afford it. Most people employed environmentally conscious methods for disposing of used electrical equipment with the majority taking it to a local authority recycling facility (63%). Some people gave their equipment away (10%), whilst a minority (5%) took their equipment apart and recycled materials. Other respondents commented that they use in-store item collection recycling services or specialist recycling services.

For the survey, we used the term ‘home healthcare robots’ (HHR) as this was a phrase which was preferred by the public involvement group. We described HHR as robots who ‘provide numerous health and care services to their patients in a variety of forms such as monitoring personal health and safety, providing medication management and scheduling, detecting people lying on the floor, assisting in physical, cognitive and occupational therapy and nursing tasks’. However, we asked the respondents to focus their responses on those which would ‘assist with physical mobility and occupational therapy type tasks’ which align with assistive robots.

#### Social norms and legal barriers to usage

Older people weren’t intending to use a robot, and concerns pertained to legal issues and errors that the robot might make were another clear barrier to adoption. The response mean averages showed that for most factors, people neither strongly agreed nor disagreed with the statements (highlighted in Table 3 in orange). However, where trends were more dominant (highlighted in Table 3 in green and red), it was clear that no-one in the individuals’ social sphere of influence were encouraging the use of assistive robots, but that if robots were used, people felt they would have the necessary means to engage with it (i.e., internet access) and that they would also need a specific support service available to help with any difficulties using the robot.

**Table 3:**
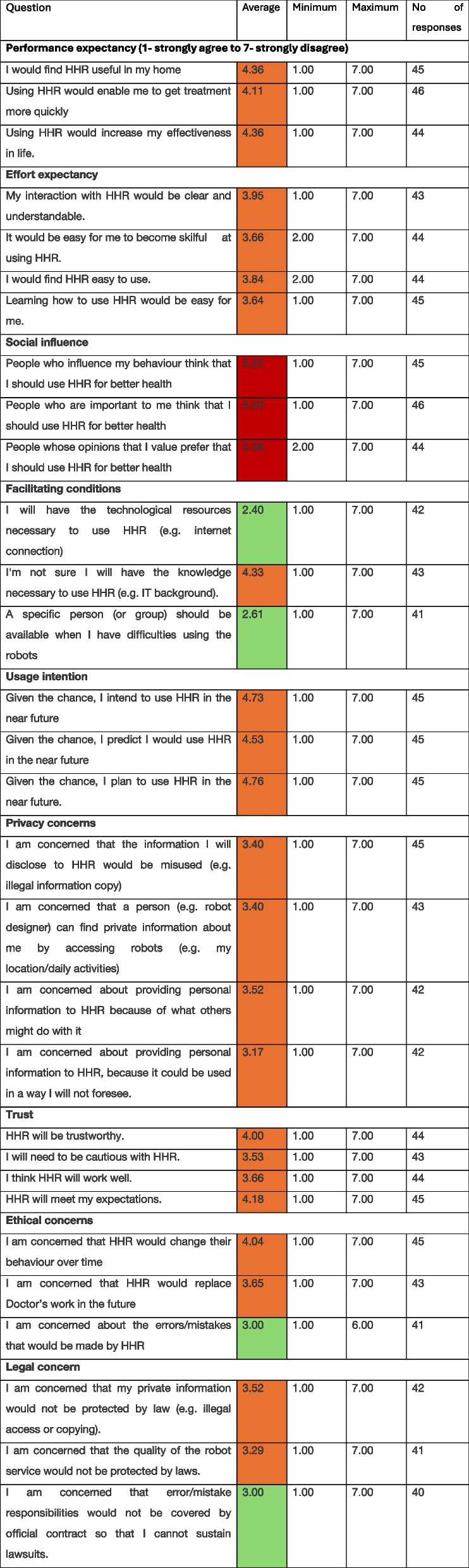
Respondents ratings of factors of interest for user acceptance of home healthcare robotics

#### Likelihood of robotic usage

Many respondents (47%) also reported that they were unsure whether they would use the robot, whilst only one respondent reported that they would not use the robot due to the high running costs.

From the robot dissection and life cycle analysis work, it was calculated that a basic home robot (i.e., TurtleBot4 as example) that could help to prevent frailty would cost on average between £1500-£2000 to buy at the current prices (2023) but is expected to be significantly lower if deployed in large scale. In terms of running costs, each charge to full battery would cost the same as boiling 1 L of water in a kettle (4 cups) or having a typical LCD TV on for 1 h (around 26p). If the robot was in constant use, it would need to be charged around 2–3 times per day (total daily charge cost around 78p). This insight was presented in the questionnaire to provide a sense of relatability and context for the participants. Based on this information, 22% stated that they would be willing to buy the robot and use it at home, 18% were willing to rent the robot, and 56% would be willing to use it if it was provided free-of-charge.

## Discussion

This paper addresses a critical issue for robotic development insofar as environmental considerations are associated with the scalability of adoption for pre-frail older populations [41]. Robotic design increasingly needs to address this considerable knowledge-to-action gap [42] particularly in relation to wider home use of robotics that depends on home-user choice. In this case the choices of pre-frail independent living adults were central and even though the survey reports only a 50% priority for the adoption based on sustainability, there are additional factors that come from the life cycle assessment indicating the need to consider a value associated with the carbon footprint and the supply-chain supporting robotic. There are advances in robotics that would come at the price of negatively impacting the environment by increasing demands on energy, contributions to greenhouse gas emissions and environmental pollution and from production to improper disposal [43]. The development of the home-based robotics repair infrastructure, has benefitted from disassembly in the lifecycle assessment. It suggests that reuse, and refabrication, in the designs are important and some authors now call for reduced complexity, self-healing, or biodegradable components to make the manufacture and adoption of devices more sustainable [44].

The results of the TurtleBot4 assessment revealed that the battery and printed circuit boards were the most significant environmental sources of impact suggesting that there is a need for more detailed evaluation. These components are critical for the circularity and sustainability of robotics in the home. Therefore, the following recommendations for improving the environmental sustainability of the robot can be summarised as: reducing replacement frequency of the battery and ensure it is sourced from more environmentally responsible suppliers [45]; these components need to be prioritised for proper disposal and best recycling methods [46]; regarding the printed circuit boards, a more in-depth understanding of their components and respective manufacturing origins is required [47], and an exploration of potential recycling options within the UK for the recovery of embedded metals like silver and gold are the initial main aspects to consider [48].

The scoping review consistently identifies that older people are able to accept the help of robots but there are new and important environmental considerations associated with the saleable manufacture and disposal of robotics. The current evidence suggests that adoption models are not focusing on this critical issue but the survey suggests that there is a great level of nuance in the way that older adult stakeholders consider sustainability and useability. A key factor in personal procurement and adoption by pre-frail older people appears to be around three themes. The first is certainly a concern for individual household financial and environmental cost of purchase, and energy usage and durability- a concern attributed to many electronic devices. The second is the legal framework for data protection as it would apply to socially assistive and assistive robots in the home- particularly associated with GDPR but also the ethical sharing of information. This finding is consistent with the new report for the Institute for Mechanical Engineers [5] that says that the demand for automated systems raises critical concerns for ethics and safety and this needs to be addressed in the design process.

The third was the reliability and function of the technology and the maintenance and the failure of the device, with the risk of poor activation and performance of the robot. Our survey also suggests that outside the research/academic setting, there is a very limited social proposition that robots are useful or usable in terms of domestic usage.

Users would prefer robots to take-over the more manual, labour-intensive tasks [49, 50] this is highlighted by two key factors: the high cost of purchase and maintenance, and the little evidence that these robots can aid human caregivers [51]. The focus on the TurtleBot4 was indicative of only some of the resource efficiencies and running costs; a separate and distinct area to now consider in design. Companies that are designing, making and selling assistive robots are not audited for their energy efficiency (as other white goods are, see Energy Saving Trust [52]) and this perhaps reflects a focus on safety in the medical devices domains that robotics would need to meet. Medical device certification does not include criteria associated with life cycle assessment or specific quality criteria for batteries or power usage [53]. The academic sector has an important role to play in the exploration of batteries, materials and enduring solutions that genuinely represent functional enhancement to the activities of daily living of older people, however this can only be achieved in the context of proper funding for development and a sustained investment in the policy to support digital applications [54] and AgeTech as evidenced in other countries [55].

Community dwelling older people regard robotics as an opportunity to undertake some of the menial tasks [56] and as purchasers and consumers of assistive devices and of care packages, they might make use of robotic technologies. The critical feature of technology research includes ‘perceived usefulness’ [56] and this is a challenge to robotic developers and computer science to undertake more innovative and transdisciplinary research. For example, products such as task reminders, communication services, fall detection, online grocery shopping, and journey planning are all more feasible on smartphones and tablets, which have seen a significant increase in uptake amongst older adults over the past decade [57]. The nature of academic research, increasingly concerned with application and real-world impact necessitates considerably more attention to social and environmental factors, to be responsive to consumers concerns [58]. Of key importance is the engagement of the end-user in project design and efforts are needed to extend the project pathways, to ‘fail faster’ and to increase the trust and transparency of innovative robotic design [59]. The findings clearly identify that the market for socially assistive robotics is highly specialised, and that task recognition and robotic execution need to be very carefully analysed before a product is tested with older people. One such example of best practice is the researchers at Trinity College Dublin who are developing a social robot for older adults using user-centred design [60]. The robotic field needs to quickly identify products and designs that have no enduring benefits and lead to storage rooms in universities, filled with robots and projects that did not go beyond the project lifespan. The perception of older people needs to be trusted and properly resourced so that experience and critical learning can be extended and drive digital literacy along with strategic planning. Incremental exposure to robots may help to bridge any gaps in technology literacy, increasing participants’ trust and enabling participants’ comfort with, and expectations of the robot to evolve [28]. The importance of staff training and reliable infrastructure, such as internet services, was also considered essential. Co-design and participatory processes help to align the product with the values and lived experience of older populations [61] and a purposeful process is essential to avoid developing apathy amongst older adults due to once more being asked to come to a focus group and not seeing a change from it.

### Strengths and limitations

- This study was conducted in association with a transdisciplinary group of researchers, looking at the adoption of robotics with older people. Allied Health Profession researchers are able to promote critical thinking associated with user need and barriers to scaling take up of devices
- This paper is the first paper known to the authors which highlights the stark gap in technology and robotic adoption literature for the consideration of environmental and sustainability factors.
- The scoping review with two follow-on studies allowed researchers to consolidate evidence from existing literature, older adults at risk of frailty and robotic life cycle assessment to understand the multidimensional complexities of technology adoption in this population group.
- The study’s scoping review only reviewed literature from 2008 onwards due to the nature of the scoping review starting from a date when smartphone technology use was prevalent which may inadvertently omit any previous literature on this topic. Additional databases could also have been searched.
- The survey only reported data from a small population group, based within a specified geographical region.
- Few participants in the survey identified with being “pre-frail” and therefore were less likely to recognise the domestic care needs they may experience. However, this may also be a barrier to technology adoption targeting the pre-frailty market as awareness of future care needs is low.
- The life cycle analysis highlighted the environmental impact of a basic robotic device. Whilst this process was robust and provided an accurate estimate of resource and energy usage, devices which fulfil the needs of older adults are likely to have higher energy demands and resource requirements.

## Conclusions

Robotics are potentially useful to people pre-frailty but the adoption of assistive and socially assistive robots for personal use in the home needs to be factored against the current and future environmental challenges. The cost, environmental impact, and availability of user-focused design may limit the scalability of deployment in personal residences. Robots have the potential to help with the caregiving and domestic needs of the growing ageing population and older adults, but robots need to be sustainably produced and have real functional benefit to be acceptable.

## Supplementary Information

Supplementary Material 1. Appendix 1: Search Strategy.

Supplementary Material 2. Appendix 2: Table of study characteristics.

## Data Availability

The datasets generated and/or analysed during the current study are available in the SHURDA repository, [http://doi.org/10.17032/shu-0000000212] (https:/eur02.safelinks.protection.outlook.com/?url = http%3A%2F%2Fdoi.org%2F10.17032% 2Fshu-0000000212&data = 05%7C02%7Ca.dinuovo% 40shu.ac.uk% 7C87af6a03147b432496b708dcfd728570 % 7C8968f6a1ac13472fb899f7316e439f43 %7C0%7C0% 7C638663915511838139% 7CUnknown% 7CTWFpbGZsb3d8eyJWIjoiMC4wLjAwMDAiLCJQIjoiV2l uMzIiLCJBTiI6Ik1haWwiLCJXVCI6Mn0 %3D%7C0% 7C%7C% 7C&sdata = oF0P% 2Fo9UNz4oVCtuNfKne51WvluYwFSvC3dUo2EMcoU %3D& reserved = 0).

## Abbreviations

AI: Artificial Intelligence
MRP: Mobile Remote Presence
NHS: National Health Service
SARs: Socially Assistive Robots
