# Supplementary material for: What social and environmental considerations are important for socially assistive robotic adoption for pre-frail older adults at home: a scoping review, life cycle assessment and survey
Source: BMC Geriatr. 2026 Feb 27;26:460. doi: 10.1186/s12877-025-06892-8 (PMC13049708; doi:10.1186/s12877-025-06892-8)
Supplement: Supplementary file 1 — Supplementary Material 1. Appendix 1: Search Strategy. [file 12877_2025_6892_MOESM1_ESM.docx]

**Search Strategy**

**Final Search Strategy**

Older Adults **AND** Community **OR** Residential **OR** Home **AND** Healthcare **AND** Robots **AND** Adoption **OR** Usage

From 2008 onwards

**Inclusion Criteria:**

- Older adults (65+ years)
- Pre-frail
- Free-living
- Home
- Community
- Robotics
- Technology
- Home
- Community
- Robotic adoption or usage
- Sustainability/environmental consideration
- Qualitative
- Quantitative
- English language

**Exclusion Criteria:**

- Non-human
- Children
- Frailty
- Surgical
- Secondary care
- Palliative
- Living in care homes/care environment
- Telemedicine/telehealth
- Internet of things
- Acute care
- Hospital care
- No primary outcome regarding robotic adoption, usage, sustainability or environment
- Reviews
- Non-English Language
