# Supplementary material for: What social and environmental considerations are important for socially assistive robotic adoption for pre-frail older adults at home: a scoping review, life cycle assessment and survey
Source: BMC Geriatr. 2026 Feb 27;26:460. doi: 10.1186/s12877-025-06892-8 (PMC13049708; doi:10.1186/s12877-025-06892-8)
Supplement: Supplementary file 2 — Supplementary Material 2. Appendix 2: Table of study characteristics. [file 12877_2025_6892_MOESM2_ESM.docx]

**IM ACTIVE – Supplementary Material**

**Detailed Characteristics of Included Studies**

| **Title** | **Author(s) and year** | **Country** | **Aim** | **Summary of Main Findings** |
| --- | --- | --- | --- | --- |
| Perspectives on a telepresence robot at an independent living facility: Lessons learned and implications | Arthanat, S., Rossignol, H., Preble, E., Grimm, K., Corvini, M., Wilcox, J., Aytur, S. and Doyle, M. (2024). | USA | To gather the perspectives of residents, trainers and staff at a retirement facility on their experience with a telepresence robot during and following a five-week wellness program. The Unified Theory of Acceptance and Use of Technology (UTAUT) served as the interview framework to explore the facilitators and barriers to the implementation of the robot. | Incremental exposure to Maxine (telepresence robot) helped to bridge any gaps in technology literacy, enabling participants’ comfort with, and expectations of, to evolve. The participants confidence and comfort in handling Maxine were increased with continued and multiple exposure and usage. It was understood that overall, user acceptance enabled the facilitation of social connections, aging in place and the delivery of health services. However, older adults still favoured the delivery of in person healthcare services and support. A hybrid model could be useful, alternating between in-person and a telepresence robot. Information technology education, staff training and reliable internet connectivity are the precursors to optimizing the value and perceived usefulness of the technology. |
| Mobile remote presence systems for older adults: Acceptance, benefits and concerns | Beer, J. M. and Takayama, L. (2011). | USA | To understand older adults' views on what a mobile remote presence system may be used for and to understand older adults perceived benefits and concerns about the system. Also, to understand criteria for older adults to accept using the system. | The older adults' opinions of the mobile remote presence system were overall positive in nature (66%), some were mixed (28%) and a few were negative (6%). The identified benefits of using the system (n = 174) significantly outweighed any related concerns (n = 124), χ² = 15.4, p < .0001. When the benefits of using technology are clear, older adults are willing to adopt its usage. The participants recognised that visualisation, reducing travel, and socialisation were primary benefits of using this system. However, etiquette of managing calls, privacy, and a lack of face-to-face contact were potential causes for concern. |
| Acceptance and perceived usefulness of robots to assist with activities of daily living and healthcare tasks. | Hall, A. K., Backonja, U., Painter, I., Cakmak, M., Sung, M., Lau, T., Thompson, H. J. and Demiris, G. (2017). | USA (Seattle, Washington Metropolitan Area) | To understand acceptance and perceived usefulness of tasks performed by robots among young, middle-aged, and older adults. | Regardless of age, participants were less comfortable with robots fulfilling the following tasks: dog walking, medical operations and elderly care. Of the 21 different tasks that were included in the survey, 8 significant differences were identified between all age groups relating to the robot's perceived usefulness in: assisting with cleaning, escorting around town, providing medical advice, delivering meals, personal care, companionship, assessing sadness and calling for help if necessary (p < 0.05). Additionally, less than half (40.2%) of older adults disclosed that robot assistance in the hospital would be acceptable or wanted (p < 0.001). Robots have the potential to help with the caregiving and domestic needs of the growing ageing population and older adults with multiple chronic conditions. |
| Are we ready for robots that care for us? Attitudes and opinions of older adults towards socials assistive robots. | Pino, M., Boulay, M., Jouen, F. and Rigaud, S. A. (2015). | France (Paris) | To clarify several aspects related to the acceptance of Socially Assistive Robots (SAR) by older adults. In particular, examining if opinions and attitudes toward SAR differed among three groups of older adults living in the community: healthy elderly individuals, persons with MCI, and informal caregivers of persons with dementia. | Participants were less likely to use the SAR at the current time (M = 0.84. SD = 0.98), but more likely to use it in the future (M = 1.96. SD = 0.88). A Wilcoxon Signed-ranks test indicated that this difference was significant (z = −3.08. p < 0.002, two-tailed test). It is a complex task to ensure that the design of SARs are both acceptable and efficient. There is no SAR configuration that fits all scenarios, systems need to be flexible and customisable to adapt. Elderly people concerned by cognitive impairment recognise the potential of SARs for supporting their health and social care at home. |
| Robots for elderly care: Review, multi-criteria optimization model and qualitative case study. | Sawik, B., Tobis, S., Baum, E., Suwalska, A., Kropinska, S., Stachnik, K., Perez-Barnabe, E., Cildoz, M., Agustin, A. and Wieczorowska-Tobis, K. (2023). | Poland (Krakow) | To perform focus group discussions to collect opinions about robot-related requirements of the elderly. | There is much interest relating to the use of robots in various aspects of care for older people. Both older people and their caregivers expressed significant demand for robots that provide broad support. Before introducing the robot to the older person, pre-training should be given that considers a wide range of ethical and practical issues. It is essential to involve the future robot users in the preparation and customisation of the technological solution to be introduced, following the actual needs and preferences of the older people themselves. |
| Robot companions and sensors for better living: Defining needs to empower low socio-economic older adults at home. | Vagnetti, R., Camp, N., Story, M., Ait-Belaid, K., Bamforth, J., Zecca, M., Di Nuovo, A., Mitra, S. and Magistro, D. (2024). | UK | To analyse and understand the perception and needs of low-income older adults regarding SARs, monitoring technologies and their use in home. | Overall, five key areas were highlighted that should be considered when designing SARs to ensure that requirements are met, and quality of life is enhanced for low-income older adults: 1) Promote and monitor an active lifestyle 2) aid with daily errands and provide physical assistance 3) reduce loneliness and isolation 4) considerations relating to monitoring technologies 5) barriers impacting SAR acceptance and usage. |
| Technology to support aging in place: Older adults' perspectives | Wang, S., Bolling, K., Mao, W., Reichstadt, J., Jeste, D., Kim, H. C. and Nebeker, C. (2019). | USA (San Diego, California) | To involve residents of local continuing care senior housing community in conversations about technologies that might facilitate their continued independent living status. Also to assess their privacy attitudes and preferences and identify residents' interest in co-designing technologies. | It was identified that the potential benefits offered by technologies were often prevented due to older adults having a lack of confidence in their ability to understand how to use or access them. It was also highlighted that inadequate software and hardware interfaces cause frustration in older adults' interactions with digital products and concerns relating to data privacy in older adults' daily usage of technology were very high (87.1%). Increasing the technology literacy of older adults can provide meaningful improvements in helping these users interact more successfully with technology. There is also a need to educate technology creators about older adults to increase the literacy of technologists. |
| Older adults actual use and adoption intention of smart health technologies in Hong Kong | Zhang, J., Wang, H., Lee, B. Y. H., Pang, M. Y. C. and Luximon, Y. (2022). | China (Hong Kong) | To investigate if the use of smart health care technologies, participants health status, sensation, and capability could be the motivators of actual use and adoption intention. | Nursing and Assistive Robots (NARs) had not been used by any of the participants. However, 62.5% of participants stated that they would be willing to use them (mean = 6.20), with 2 participants being unwilling and one participant not reporting adoption intention due to having never seen this type of product. Results suggest that gender, age, living situation, cognitive capabilities and educational level can all influence the adoption intention. |
